# Selective Targeting of the L858R Mutation (EGFR) in Non-Small Cell Lung Cancer: A Mechanism for Advancing Targeted Chemotherapy

**DOI:** 10.3389/fonc.2017.00104

**Published:** 2017-05-30

**Authors:** Rohan Arora, Venkat Krishnan

**Affiliations:** ^1^American High School, Fremont, CA, United States

**Keywords:** lung cancer, EGFR, targeted therapy, small molecule–cytotoxin conjugate, L858R

## Abstract

Lung cancer remains one of today’s most deadly and intractable cancers. Non-small cell lung cancer (NSCLC) accounts for roughly 85% of lung cancers, with an extremely poor survival rate. To ensure patient comfort and survival, the development of a selective therapy is imperative. However, lung cancer does not display surface proteins associated uniquely with tumor cells; thus, it is very difficult to develop a tumor-specific drug. Current techniques that target overexpression of proteins or inhibit growth pathways are either non-specific or prone to rapid drug resistance. The goal was to design a drug targeted to structural mutations expressed by tumor-associated general surface proteins, thereby combating the lack of tumor-unique markers in lung cancer. Mutant EGFR was identified as a potential target due to its prominence in tumor cells. Due to their size, it was determined that small molecules would be most effective at targeting isolated changes in protein structure, and thereby differentiating between the tumor-associated mutant EGFR and the healthy wild type. Conformational analysis of a virtual binding study conducted in VINA predicted a set of drug-like small molecules specific for the L858R mutation in EGFR. One molecule (ZN47) was then acquired and conjugated to a carrier protein to form a multifaceted hapten–protein conjugate. Multiple ELISAs were conducted to confirm the specificity of the conjugate to both tumor-associated mutant EGFRs. The results indicate that the identified molecule may be highly selective for tumor-associated L858R-EGFR, but further research, including a complete dosage-binding study, is necessary for full validation.

## 1 Introduction

Lung cancer accounts for 27% of cancer deaths in both men and women, making it by far the most deadly cancer. Of all lung cancer cases, approximately 85% are categorized as non-small cell lung cancer (NSCLC), which includes adenocarcinoma, squamous cell cancer, and large cell cancer. It is often fatal and has a Stage IA survival rate of 49%, which drops to 1% as the cancer metastasizes (Stage IV) (1). NSCLC and other cancers are commonly treated with a variety of targeted therapeutics that aim primarily to inhibit key growth mechanisms (2). For example, FDA-approved treatments such as erlotinib and gefitinib inhibit the function of the epidermal growth factor receptor (EGFR) and have been shown to be most effective with NSCLC-associated mutations of EGFR. Although they are relatively successful, these drugs have a variety of limitations and drawbacks, including some degree of non-specificity, as well as frequently acquired resistance (3).

Highly selective therapy is widely recognized as critical to the successful treatment of various cancers (4). Although a variety of EGFR tyrosine kinase inhibitors (TKIs) have been released or are being researched as targeted therapeutics for NSCLC, the presence of a widely acknowledged targeted chemotherapeutic is lacking. EGFR TKIs are effective at slowing the proliferation of a tumor cell, but do not lead directly to the death of the cell (5). Further, they are often non-specific, causing a series of side effects, and are prone to drug resistance (6). A targeted mechanism for cytotoxin delivery is necessary to advance the treatment of NSCLC and increase the comfort and survival of patients.

Traditional targeted cytotoxins require the presence of a tumor cell-unique surface protein to serve as the binding site for an antibody vector (7). There is no such protein found across a large percentage of NSCLC cells, making it exceptionally difficult to develop a targeted chemotherapeutic capable of selecting only cancer cells (8). However in NSCLC, as well as in various other cancers, tumor cells do commonly express slightly mutated versions of common surface proteins, such as EGFR (9). We hypothesized that these could potentially be exploited as molecular targets for selective cytotoxin delivery.

The objective of this current study was to develop a molecular vector that successfully recognizes and selectively binds to the L858R mutation of EGFR found in NSCLC cells, thus differentiating between tumor and healthy cells. As traditional antibodies would remain too large to differentiate on the basis of an individual mutation, it was determined that the best such vector would be in the form of a small molecule.

## 2 Materials and Methods

A database of 7 million drug-like small molecule structures partitioned into groups by similarity was downloaded from ZINC. A “diversity set” was created for initial screening by selecting 19 molecules at random from each of the 2,000 groups. Each of the 38,000 small molecules were screened against both the wild-type (RCSB: 1IVO) and mutant receptor files (10), and information on the affinity and confirmation (binding residues) was collected for each screening. A single molecule was identified to be selective *in silico* for the mutant on the basis of binding conformation.

**Figure 1:**
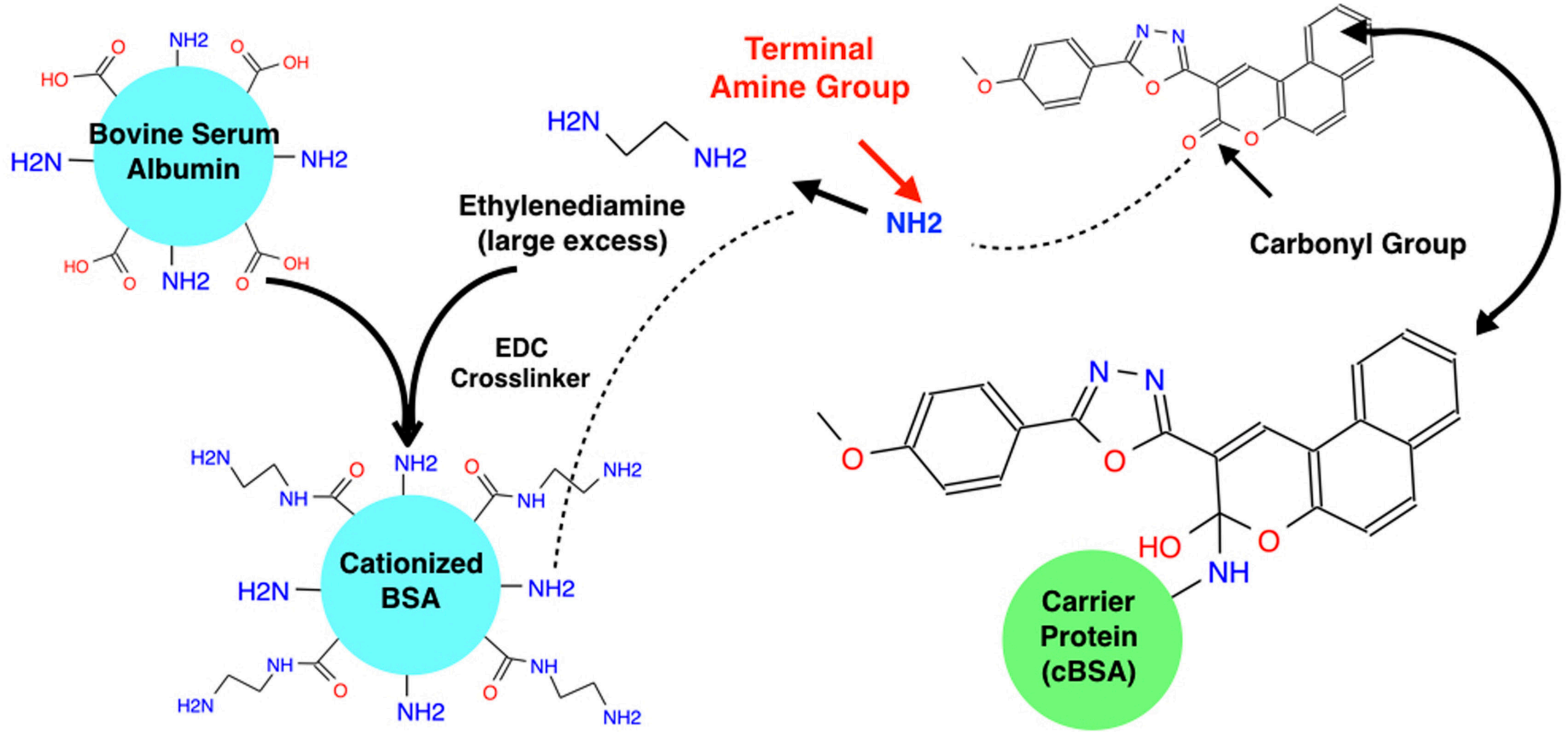
**Mannich reaction to conjugate hapten and BSA carrier protein**. The carbonyl Group on ZN47 is not involved in any interaction with the L858R-EGFR mutant and thus when it is broken to attach the carrier protein, it does not affect ZN47’s binding with the L858R-EGFR mutant. A Mannich reaction is used to break ZN47’s carbonyl group and break a hydrogen from the carrier protein’s terminal amine group which attaches to the single bonded oxygen from the broken carbonyl group on ZN47 to form a hydroxyl group. The NH from the carrier protein attaches to the alpha-carbon group on ZN47, linking ZN47 to BSA.

The small molecule was then obtained from Life Chemicals for *in vitro* testing. A binding study was developed and conducted. After *in silico* conformation (using PyMol and VINA) that conjugation would not affect the vector’s original binding affinity and conformation, the small molecule was first conjugated to a carrier protein (biotin-bovine serum albumin) with a Mannich reaction (Figure 1). This resulted in the covalent bonding of an amine group on the ethyldiamine linker and an inactive carbonyl group on the molecule. (The conjugation was confirmed with Native page gel-electrophoresis.) All reagents (desalting columns, purification buffer salt, an aldehyde, MES buffer, and Biotin BSA) were ordered from Thermo Fisher Scientific. The Imject PharmaLink Immunogen Kit Protocol was followed for this conjugation; however, the kit was not available so the reagents were ordered individually.

A series of ELISA assays were then conducted to evaluate the selectivity of the conjugate for L858R-EGFR. A general ELISA protocol was obtained from Abcam and followed. Standard reagents (i.e., wash buffer, assay buffer, etc.) were ordered from Thermo Fisher and stored according to instructions. A total of 20 μg of EGF-Biotin was ordered from Thermo Fisher, stored at −20°C, and diluted to 2.5 ng/ml in standard assay buffer before use. A stock solution at a concentration of 2 mM was created from the conjugate and further diluted in series to produce concentrations of 1, 0.50, 0.1250, and 0.0625 mM in purification buffer. Both forms of EGFR were ordered from Thermo Fisher, stored at −80°C in dry ice, and diluted to 3 μg/ml for binding to the plates. EGFR (WT) was diluted to 2.5 ng/μl before use in the assay. The biotinylated conjugate was tested against both the bound WT and bound L858R. The resulting absorbance was measured at 450 nm, with Streptavidin-HRP as the detecting enzyme. To simulate the potentially inhibitory effects of dimerization and activation of EGFR, free EGFR (WT) was included in solution as a competing molecule in assays with bound EGFR WT, while free EGF was included with bound EGFR-L858R.

## 3 Results

The virtual binding study indicated the small molecule ZINC4070447 (abbreviated ZN47) would successfully differentiate between the mutant EGFR and WT EGFR. Table 1 summarizes some properties of the molecule.

**Table 1:** **ZN47 molecule properties and residue interactions**.

Molecule (ZINC ID)	ZINC000004070447
Molecular formula	C_22_H_14_N_2_O_2_

Structure	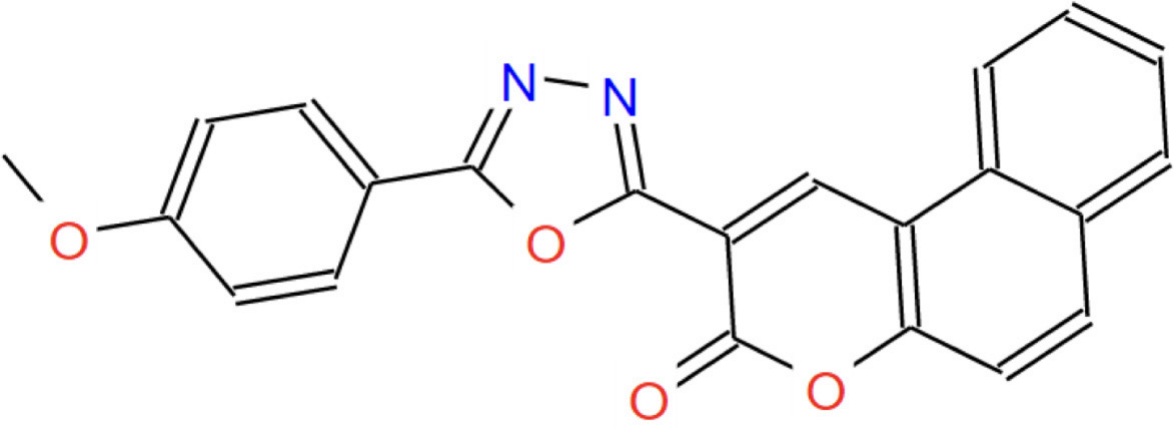

Residue interactions on receptor	*L858R*: Arg144, Leu147, Thr93, Asp158, Lys49. Asn145
	*Wild type*: Thr250, Try44, Ile 318, Arg285, Ala286, Gln252

Binding energy (kcal/mol)	*L858R*: −9.8 kcal/mol
	*Wild type*: −9.8 kcal/mol

*The table shows the properties of ZN47 obtained from in silico screening. It describes the structure, residues of interaction, and affinity of the molecule to each receptor*.

The key results from 4 separate ELISA assays are presented here to validate the results from the virtual binding study.

**Figure 2:**
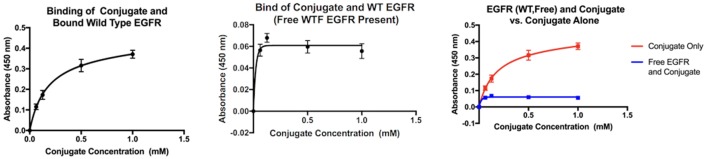
**Interaction of the MOI with wild-type EGFR alone (left) and in the presence of free wild-type EGFR (center)**. Comparison of both interactions (right).

The graphs in Figure 2 compare the interaction between wild-type EGFR and the molecule of interest both alone and in the presence of free EGFR. When alone in solution with bound wild-type EGFR (right), the absorbance values indicate that as the concentration of the conjugate increases, the level of biotin present in the well after washing increases as well. However, when free EGFR is present (center), in solution along with the MOI, the absorbance values are relatively stagnant, and the concentration of the MOI has no effect on the absorbency measured. If the two graphs are compared (right), it is clear that the absorbency measured when the MOI is alone in solution is much higher than when free EGFR is also present.

**Figure 3:**
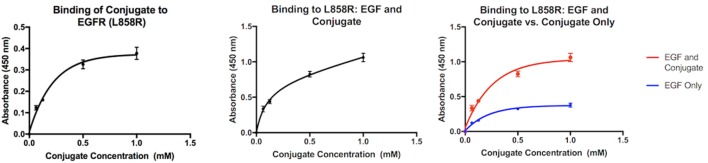
**Interaction of the MOI alone with mutant EGFR (left) and in the presence of biotin-EGF (center)**. Comparison of the two interactions (right).

The graphs in Figure 3 compare the interaction between mutant EGFR and the MOI both alone and in the presence of EGF. When alone in solution with the bound mutant EGFR, the absorbance values indicate that as the concentration of the conjugate increases, the level of biotin present in the well after washing increases as well. When EGF is present in addition to the MOI in solution (center), the same trend is observed. However, when the two interactions are compared side by side (right), it is clear that the absorbencey observed when both Biotin-EGF and the MOI are present is much higher, and more biotin is present in the second interaction.

## 4 Discussion

Virtual simulations of small molecule binding to receptors predict that ZN47 should bind to mutant L858R-EGFR over EGFR-WT, based on binding conformation. Although the identified small molecule has the same predicted equilibrium disassociation constant (kd) of 5.9 × 10^−8^ M for both receptors, conformational analysis predicted that EGFR dimerization should inhibit it from binding to EGFR-WT.

**Figure 4:**
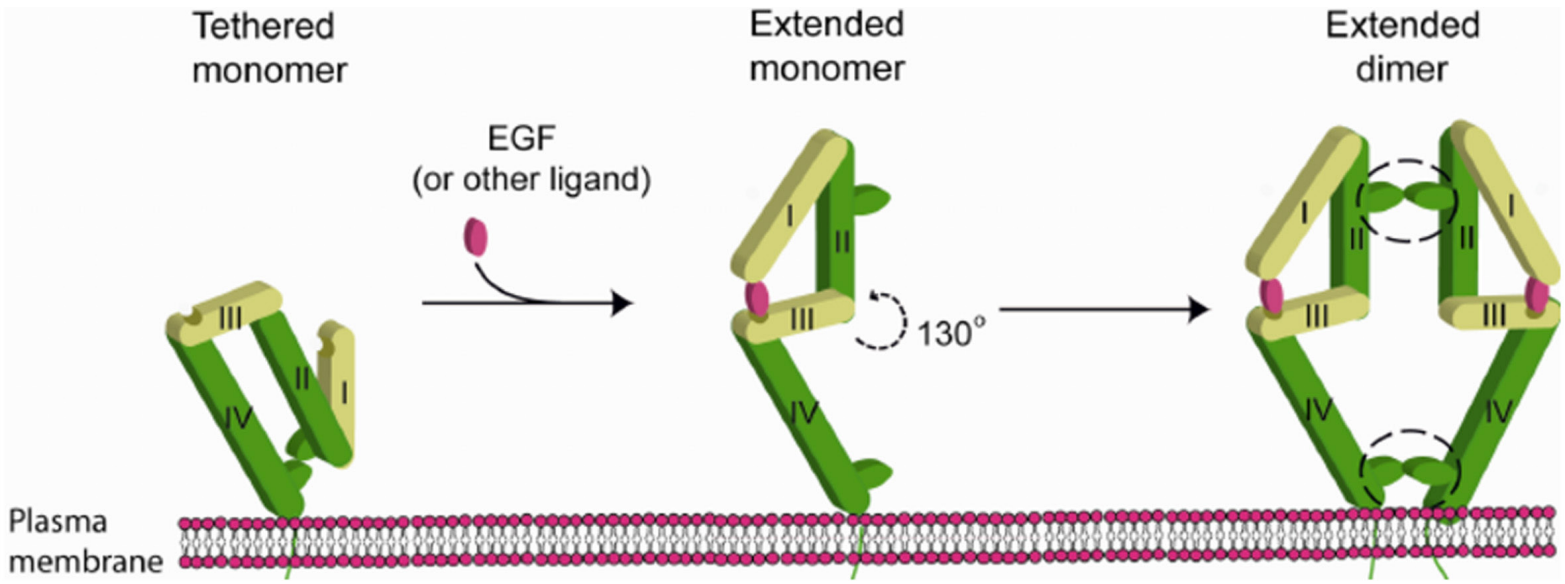
**Mechanism of EGFR activation by EGF [figure from Nevo (11)]**.

Binding of EGF to EGFR triggers receptor-based dimerization which is necessary to trigger the downstream signaling pathway. Prior to activation a critical “dimerization arm” found on Domain II is buried in an intramolecular “tether” by interacting with Domain IV within the same molecule, which auto inhibits dimerization. EGF bridges two distinct binding sites on Domain I and III when it binds to the receptor, changing the configuration so that the “dimerization arm” is now exposed. The homo-/hetero dimerization of the receptor is therefore induced and occurs primarily along the face of Domain II (but also involves interactions in Domain IV) (Figure 4).

According to literature, the extracellular domain of EGFR consists of residues Leu 25—Thr 638. Of these the dimerization arm of EGFR lies from residues 242 to 259 (12). Conformational analysis of screening results predicted that that ZN47 should bind to EGFR-WT on residues that overlap with the dimerization arm. Thus, EGFR dimerization should inhibit ZN47 binding site with EGFR-WT. *In silico* predictions show that ZN47 should bind to Domain I on L858R-EGFR (mutant), which always exposed and remains unaffected by EGFR dimerization. Therefore, ZN47 should bind to EGFR-L858R and not interact with EGFR-WT.

An ELISA assay was developed to test the assumption that dimerization would inhibit any interactions between the MOI and the wild-type by observing the interaction in the presence of free EGFR. Since the MOI interacts with mutant EGFR at Domain I, there is some possibility that it could be inhibited by EGF (which has a binding site on the same domain); therefore, a second assay was developed to verify the MOI would successfully bind to the mutant EGFR by observing the interaction in the presence of EGF.

ZN47 was then bound covalently to Biotin BSA to allow for detection in an ELISA assay and simulate a small molecule–drug conjugate. The ELISA assay results indicate that the conjugate does possess the ability to differentiate between the mutant EGFR and the wild-type, as predicted. When free EGFR (wild-type) is present in solution with the MOI and bound EGFR (wild-type), the absorbency values drop significantly, indicating that biotin is no longer present. As the MOI is the only biotinylated compound in the assay, the results show that due to the presence of free EGFR, the MOI does not bind to the bound EGFR-WT. The dimerization process triggered by free EGFR may be inhibiting the interaction, as suggested by the virtual study. When the MOI is present alone in solution with the mutant EGFR, the absorbency of the solution increases as the concentration of the MOI increases, indicating binding. When biotin-EGF is added to the solution, the same trend is observed. When the two interactions are compared, it is immediately apparent that the presence of biotin-EGF in the interaction significantly increases the final absorbency values. A higher level of biotin is therefore present when both biotin-EGF and the MOI are present than just the MOI. This can be justified if both the EGF and MOI bind to the bound mutant EGFR independently at separate locations, which explains the presence of excess biotin in the second interaction.

Although the results indicate that the MOI may have high selectivity toward the tumor-associated L858R-EGFR, marking it as a potential molecular vector, a variety of limitations exist that must be addressed before any significant conclusions can be made. In the case that future work with this specific molecule is not successful, the flexibility of the procedure ensures that a second molecule from the ZINC database can be identified and experimented with in the same manner.

### 4.1 Limitations

First, the results presented form only a portion of a complete dosage-binding study, which is necessary to determine the maximal and minimal response induced by the MOI as well as the EC50 and *in vitro Kd values*. Before cell line studies and *in vivo* research can be conducted, such a study must first be completed. The results from the ELISA study are not rigorous and must be taken as indications of success, but more data from more trials are required before absolute validation of the MOI’s selectivity can occur. Further, a control must be run to verify that the presence of free EGFR does not effect the interaction of the MOI with bound L858R mutant. Although the *in silico* predictions indicate this should not occur, it must be verified *in vitro*. The same must be done for free EGF in the interaction between wild-type EGFR and the MOI.

## Author Notes

This manuscript was originally created as a submission into the Siemens Competition, a national competition designed to provide opportunities and accolades for high school students conducting research in various fields of science, technology, engineering, and mathematics.

## Author Contributions

Both authors contributed equally to the research. VK provided technological expertise regarding the simulations, while RA contributed biological expertise during the experimental assays. However, all components of the research were completed by both authors.

## Conflict of Interest Statement

The authors declare that the research was conducted in the absence of any commercial or financial relationships that could be construed as a potential conflict of interest.
